# Safety, Mortality, and Hemodynamic Impact of Patients with MitraClip Undergoing Left Ventricular Assist Device Implantation

**DOI:** 10.1007/s12265-021-10178-w

**Published:** 2021-10-28

**Authors:** Henrik Fox, Takayuki Gyoten, Sebastian V. Rojas, Marcus-André Deutsch, René Schramm, Volker Rudolph, Jan F. Gummert, Michiel Morshuis

**Affiliations:** 1 Clinic for Thoracic and Cardiovascular Surgery, Herz- Und Diabeteszentrum NRW, Ruhr-Universität Bochum, Bad Oeynhausen, Germany; 2 Heart Failure Department, Herz- Und Diabeteszentrum NRW, Ruhr-Universität Bochum, Bochum, Bad Oeynhausen, Germany; 3 Clinic for General and Interventional Cardiology/Angiology, Herz- Und Diabeteszentrum NRW, Ruhr-Universität Bochum, Bochum, Bad Oeynhausen, Germany

**Keywords:** Heart failure, Left ventricular dysfunction, HeartMate 3, MitraClip, CentriMag system, Left ventricular assist device, Mechanical circulatory support, Mortality, Pulmonary artery pressure, Pulmonary vascular resistance, Transpulmonary gradient, Heart transplantation

## Abstract

The interactions and hemodynamic impact of transcatheter percutaneous mitral valve repair (TMR) have not yet been investigated in patients undergoing left ventricular assist device (LVAD) implantation, but hemodynamic adverse effects are feared in the combination of TMR and LVAD for altered mitral valve flow. This study investigated the hemodynamic interplay in combination of TMR and LVAD in 119 patients, and propensity score match analysis revealed no difference in both perioperative mortality and 2-year follow-up survival (*p* = 0.84). Nonetheless, postoperatively mean pulmonary arterial pressure, pulmonary capillary wedge pressure, and cardiac index improved, and multivariable cox regression analysis at 2 years identified preoperative total bilirubin and temporary right ventricular mechanical circulatory support as independent risk factors for all-cause mortality but not TMR. Prior TMR has no impact on mortality or cardiovascular complications in patients with LVAD.

**Graphical abstract:**

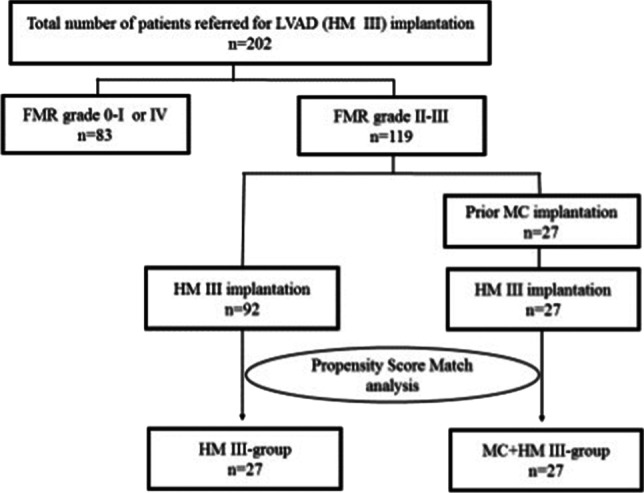

## Clinical Relevance

### What Is Known

MitraClip procedure and LVAD implantation are increasingly performed to improve symptoms but also survival in end-stage heart failure. Nevertheless, in this context, nothing is known about the interplay of MitraClip and LVAD therapy, and rumors proclaim that prior MitraClip may impair LVAD inflow and decrease LVAD therapy effectiveness.

### What Is New in This Study

This is the first study to analyze the impact of MitraClip in patients with LVAD in a clinical, but also 2-year follow-up setting, including invasive right heart catherization measurements and mortality. Our findings are substantiated through propensity score–matched analysis in the largest cohort investigated yet. We demonstrate safety of prior MitraClip implantation in patients undergoing LVAD implantation, and our results proclaim that MitraClip is not associated with worse outcome when LVAD is implanted, and we can show positive influence of LVAD regarding right heart hemodynamics encouraging colleagues to consider heart transplantation in these patients.

### What Is Next

Patients with prior MitraClip can unscrupulously undergo LVAD implantation, but further trials will have to validate significance of functional mitral regurgitation treatment prior to LVAD implantation in end-stage heart failure.

## Introduction

Functional severe mitral regurgitation (FMR) is frequently observed in patients with advanced heart failure (HF). FMR in HF patients is common, and it is not only associated with increased HF symptoms, but also poor clinical outcome [1–3]. Conventional mitral valve surgery often is a high-risk procedure in HF patients with FMR, and transcatheter percutaneous edge-to-edge mitral valve repair (MitraClip system, Abbott Vascular, Menlo Park, CA) (TMR) is considered an alternative in symptomatic patients [2, 4]. A recently published clinical trial showed that TMR in HF patients may be superior to optimal medical therapy (OMT) regarding survival and hospitalization frequency [4]. However, conflicting data are available, and retentions are expressed in HF patients after TMR that require left ventricular assist device (LVAD) implantation for HF progression [5]. It has been hypothesized that TMR may restrict mitral valve mobility, which could increase transvalvular gradients, and this could interfere or even impair LVAD inflow and LVAD function [6]. Despite this single-center and small patient cohort study, only little is known about the interplay of TMR and subsequent LVAD implantation so far, and this relationship has not yet been studied in detail. In addition to that, there is no data available on complication and mortality rates in patients treated with the combination of TMR and LVAD to date.

This study is the first trial to investigate pre-, peri-, and postoperative hemodynamic parameters as well as clinical outcomes in LVAD HF patients with and without TMR.

## Methods

### Study Design and Follow-up

Data was retrieved from our prospectively maintained patient database, and this single-center study was approved by our Institutional Ethics Committee of Ruhr-University Bochum in Bad Oeynhausen, Germany. Informed consent has been obtained between 2016 and 2020 from all end-stage HF patients with severe functional mitral valve regurgitation (FMR) in our hospital that additionally received LVAD implantation with the HeartMate 3 (HM3) device (Abbott, Abbott Park, IL, USA). This is an observational prospective study, and medical decision for either TMR or LVAD implantation was made in the heart team independently from study participation. We conducted an all-comer study; all patients with TMR were enrolled; there were no study exclusion criteria for elevated mitral valve gradient after TMR. During HM3 implantation, no patient had TMR removal or explantation.

Patients are divided into two groups: HF patients with FMR and HM3 implantation without TMR (group 1) and patients with HM3 and TMR (group 2). Patients with mitral valve stenosis without TMR (mean mitral valve gradient > 5 mmHg), surgical mitral valve repair, or mitral valve replacement were excluded. Patients with mitral valve surgery simultaneously during LVAD implantation were also not considered. Follow-up data of clinical status, survival, and cardiovascular events were collected, including transplantation, death, stroke, arrhythmia requiring cardioversion, and VAD-related complications, and follow-up is completely available in all study patients enrolled. The clinical follow-up was closed after 2-year follow-up, and endpoints of this study were predefined as all-cause death or heart transplantation.

### Left Ventricular Assist Device Implantation: Surgical Procedure

All procedures were performed by experienced board-certified cardiovascular surgeons via median sternotomy or left thoracotomy. Concomitant surgical procedures included aortic valve replacement, tricuspid valve repair, ASD closure, or resection of left atrial appendage. Cardio-pulmonary bypass (CPB) was established in standard fashion through direct cannulation of the ascending aorta and both caval veins or the right atrium in the sternotomy approach or via left groin vessels for left thoracotomy approach. Warm blood cardioplegia was applied into the aortic root, and cross-clamping was used in cases with concomitant aortic valve replacement. All other procedures were performed in beating-heart technique.

In all patients, intraoperative transesophageal echocardiography (TEE) was performed to ensure correct inflow cannulation position, and the cannula was placed in dumpling position to allow unobstructed LVAD inflow. The outflow cannula was anastomosed with an end-to-side running suture to the ascending or descending aorta using side-clamping in both sternotomy and left thoracotomy approach, respectively.

Temporary mechanical circulatory support (MCS) for right heart failure was implemented by percutaneous cannulation of a femoral vein and anastomosis of an 8-mm vascular graft (Hemashield, Getinge Gothenburg, Sweden) to the pulmonary trunk, both connected via heparin-coated standard tubing using CentriMag system (Abbott, Abbott Park, IL) with or without oxygenator (Eurosets, Medolla, Italy).

### Interventional Transcatheter Valve Repair Procedure: MitraClip Implantation

All TMR procedures were performed prior to LVAD implantation. Only patients meeting accepted and established published TMR criteria were eligible [2, 4]. The institutional multidisciplinary heart team (including cardiologists, cardiac surgeons, perfusionists, heart failure specialists, and cardiothoracic anesthesia) reviewed all indications and decided on every therapeutic approach individually based on influencing factors, such as age, calculated surgical risk, and cardiac and non-cardiac comorbidities, as well as mitral valve anatomy assessed by TEE. All interventional TMR procedures were performed by experienced interventional cardiologists. Clips applied (arm length 9 mm) were utilized according to established standard practice as recommended by the manufacturer in shallow sedation, guided by TEE and fluoroscopy. Residual mitral valve regurgitation had to be below moderate in TEE grading, at a mean blood pressure of ≥ 60 mmHg, to be accepted as an effective procedure. Additional clips were placed, or clips were repositioned until this requirement was met in all patients.

### Statistical Analysis

Results are expressed as mean ± standard deviation (SD) or as median + 25th to 75th percentile interquartile range for continuous variables and frequency and percentage for categorical variables. Univariable comparisons were performed using Student’s paired or unpaired *t* test for continuous normally distributed data. The Mann–Whitney *U* test was used for comparisons of non-parametric continuous data and Fisher’s exact test for categorical data. Survival and freedom from all-cause death were derived using the Kaplan–Meier method, and comparisons were made using the log-rank test. Patient characteristics of both the HM3 and TMR + HM3 group were further analyzed using propensity score match (PS) analysis with adjustments for the 5 major characteristics known to confound mortality in LVAD patients. Patients of the HM3 group were matched in a one-on-one basis to patients of the TMR + HM3 group through practice of propensity score calculations using the nearest-neighbor match without replacement, within a matching tolerance (caliper) of 0.20 and an absolute standardized difference of ≦ 10%. The rates of freedom from all-cause death in the matched cohort were generated using the Kaplan–Meier method, and comparisons were made using the stratified log-rank test. To estimate independent effects regarding all-cause death, Cox proportional-hazards regression analysis (also stratified on matched pairs) was subsequently applied to the matched population to identify independent predictors of mortality. Candidate covariates were chosen based on current medical knowledge. A *p* value of < 0.05 was considered statistically significant, and all reported *p* values are two-sided. All statistical analyses were performed using R.

## Results

### Baseline Patient Characteristics and Perioperative Parameters

A total of 119 patients with FMR undergoing HeartMate 3 LVAD implantation were identified for inclusion in this study during 2016 and 2020. All demographics and clinical characteristics are summarized in Table 1. Study patients were divided into two groups: patients without TMR (group 1, *n* = 92) and patients with TMR (group 2, *n* = 27) (Table 1). Patients in group 2 were marginally older (*p* = 0.050) and had worse left ventricular ejection fraction (LVEF, *p* = 0.035), while all other baseline characteristics revealed no statistically significant differences and therefore allow comparability of the two study groups (Table 1). Etiology of HF was ischemic in *n* = 47 patients (51%) and non-ischemic in *n* = 45 patients (49%) in group 1, as well as ischemic in *n* = 19 patients (70%) and non-ischemic in *n* = 8 patients (30%) in group 2 (*p* = 0.084). At the time of LVAD implantation, the mean distance from TMR was 26 months (3.5–26; range, 0–85), and distribution of the Interagency Registry for Mechanically Assisted Circulatory Support (INTERMACS) Profile levels were comparable in both groups (*p* = 0.76). All patients underwent coronary angiography prior to LVAD implantation to assess coronary artery status, and only one patient in group 1 required concomitant coronary artery bypass graft surgery.

**Table 1:** Baseline characteristics, results of transthoracic echocardiography, and right heart catheterization of the full cohort and PS-matched cohort; *n* (%) if not otherwise specified

Characteristics	Unmatched cohort			PS-matched cohort		
	HM III + FMR (*n* = 92)	HM III + TMR (*n* = 27)	*p* value	HM III + FMR (*n* = 27)	HM III + TMR (*n* = 27)	*p* value
Age, mean ± SD (years)	58 ± 12	63 ± 9.1	0.05	64 ± 5.5	63 ± 9.1	0.72
Male gender	80 (87)	23 (85)	0.73	25 (93)	23 (85)	1
Body mass index, mean ± SD (kg/m^2^)	27 ± 4.7	27 ± 4.3	0.76	28 ± 4.5	27 ± 4.3	0.41
Arterial hypertension	48 (52)	18 (67)	0.2	20 (74)	18 (67)	0.77
Diabetes mellitus	22 (24)	8 (30)	0.62	10 (37)	8 (30)	0.77
COLD	14 (15)	3 (11)	0.76	5 (19)	3 (11)	0.7
PAD	10 (11)	4 (15)	0.52	4 (15)	4 (15)	1
Stroke	13 (14)	9 (33)	0.045	2 (3.7)	9 (33)	0.039
Heart failure etiology						
Ischemic heart failure	47 (51)	19 (70)	0.084	18 (67)	19 (70)	1
Non-ischemic heart failure	45 (49)	8 (30)	0.084	9 (33)	8 (30)	1
Previous CRT	29 (32)	17 (63)	0.0062	10 (37)	17 (63)	0.1
Previous ICD	69 (75)	27 (100)	0.0018	21 (78)	27 (100)	0.023
Number of MitraClips implanted	-	1.8 ± 0.64	-	-	1.8 ± 0.64	-
INTERMACS	2.5 ± 0.93	2.5 ± 0.94	0.76	2.7 ± 0.83	2.5 ± 0.94	0.54
Level 1	14 (15)	3 (11)		2 (7.4)	3 (11)	
Level 2	36 (39)	12 (44)		9 (33)	12 (44)	
Level 3	28 (30)	7 (26)		12 (44)	7 (26)	
Level 4	14 (15)	5 (19)		4 (15)	5 (19)	
Laboratory parameters						
Blood urea nitrogen	67 ± 41	70 ± 46	0.75	79 ± 43	70 ± 46	0.48
Creatinine	1.5 ± 0.78	1.5 ± 0.55	0.97	1.5 ± 0.49	1.5 ± 0.55	0.7
T-bilirubin	1.8 ± 2.3	1.6 ± 1.1	0.72	1.5 ± 1.2	1.6 ± 1.1	0.82
Echocardiography						
LVEF mean ± SD (%)	22 ± 6.1	19 ± 4.9	0.035	22 ± 6.2	19 ± 4.9	0.054
LVDd, mean ± SD (mm)	70 ± 10	72 ± 6.1	0.37	69 ± 11	72 ± 6.1	0.18
LVDs, mean ± SD (mm)	64 ± 9.9	68 ± 7.6	0.23	65 ± 11	68 ± 7.6	0.4
RVDd, mean ± SD (mm)	45 ± 9.7	43 ± 7.8	0.44	46 ± 10	43 ± 7.8	0.41
LVEDV, mean ± SD (ml)	281 ± 125	317 ± 100	0.31	267 ± 141	317 ± 100	0.27
LVESV, mean ± SD (ml)	205 ± 89	246 ± 86	0.12	198 ± 107	246 ± 86	0.18
MR grade, mean ± SD	2.5 ± 0.52	2.3 ± 0.68	0.28	2.3 ± 0.47	2.3 ± 0.68	0.82
TR grade, mean ± SD	1.8 ± 0.76	1.8 ± 0.72	0.79	1.9 ± 0.75	1.8 ± 0.72	0.56
TAPSE, mean ± SD (mm)	17 ± 5.5	17 ± 4.8	0.94	18 ± 5.6	17 ± 4.8	0.84
MVPG, mean ± SD (mm)	-	2.44 ± 0.95	-		2.44 ± 0.95	-
Right heart catheterization						
Systolic PAP, mean ± SD (mmHg)	51 ± 15	50 ± 13	0.92	59 ± 13	50 ± 13	0.037
Mean PAP, mean ± SD (mmHg)	36 ± 10	34 ± 8.0	0.35	41 ± 9.1	34 ± 8.0	0.0071
Diastolic PAP, mean ± SD (mmHg)	28 ± 9.0	26 ± 6.2	0.39	31 ± 8.2	26 ± 6.2	0.018
PCWP, mean ± SD (mmHg)	27 ± 9.2	25 ± 8.2	0.35	31 ± 7.9	25 ± 8.2	0.014
SVR, mean ± SD (dynes/sec/cm^−5^)	1883 ± 722	1665 ± 585	0.23	1764 ± 581	1665 ± 585	0.58
PVR, mean ± SD (dynes/sec/cm^−5^)	274 ± 191	237 ± 138	0.4	303 ± 246	237 ± 1 38	0.28
CO, mean ± SD (L/min)	3.3 ± 2.2	3.5 ± 1.1	0.7	3.2 ± 0.95	3.5 ± 1.1	0.34
CI, mean ± SD (L/min/m^2^)	1.5 ± 0.41	1.7 ± 0.48	0.11	1.6 ± 0.48	1.7 ± 0.48	0.45
CVP, mean ± SD (mmHg)	14 ± 6.6	15 ± 5.4	0.68	15 ± 6.2	15 ± 5.4	0.86
DPG, mean ± SD (mmHg)	8.9 ± 4.2	9.3 + 3.0	0.7	10 ± 4.5	9.3 ± 3.0	0.41
TPG, mean ± SD (mmHg)	10 ± 6.3	9.4 ± 5.3	0.69	11 ± 6.3	9.4 ± 5.3	0.52

*HM*, HeartMate 3; *TMR*, transcatheter percutaneous mitral valve repair; *COLD*, chronic obstructive lung disease; *PAD*, peripheral artery disease; *CRT*, cardiac resynchronization therapy; *ICD*, implanted cardioverter defibrillator; *INTERMACS*, Interagency Registry for Mechanically Assisted Circulatory Support; *LVEF*, left ventricular ejection fraction; *LVDd*, left ventricular diastolic diameter; *LVDs*, left ventricular systolic diameter; *RVDd*, right ventricular diastolic diameter; *RVDs*, right ventricular systolic diameter; *LVEDV*, left ventricular end-diastolic volume; *LVESV*, left ventricular end-systolic volume; *MR*, mitral valve regurgitation; *TR*, tricuspid valve regurgitation; *TAPSE*, tricuspid annular plane systolic excursion; *MVPG*, mitral valve pressure gradient; *PAP*, pulmonary artery pressure; *PCWP*, pulmonary capillary wedge pressure; *SVR*, systemic vascular resistance; *PVR*, pulmonary vascular resistance; *CO*, cardiac output; *CI*, cardiac index; *CVP*, central venous pressure; *DPG*, diastolic pressure gradient; *TPG*, transpulmonary pressure gradient.

### Propensity Score (PS) Matching Analysis Results

**Fig. 1:**
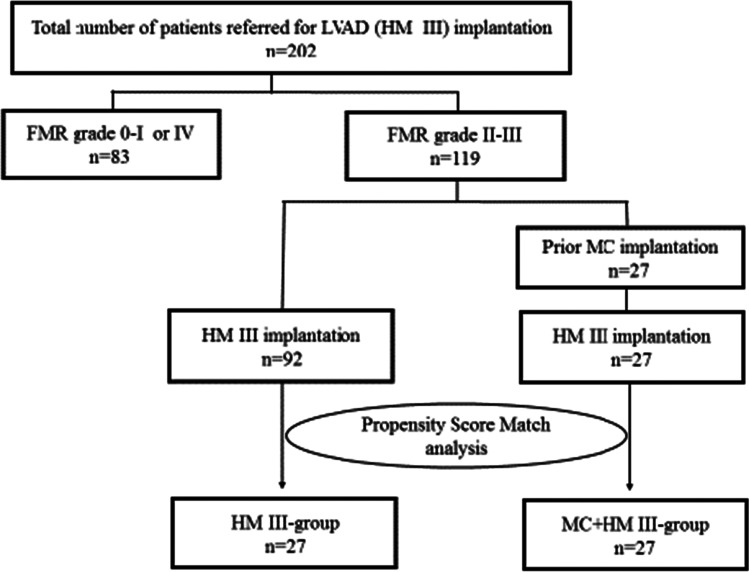
Graphical abstract: study flow diagram

To minimize potential confounding effects of selection bias and to decrease variability of both groups, we performed propensity score (PS) matching for the two groups FMR + HM3 and TMR + HM3 for clinical characteristics. PS matching was executed for age, sex, body mass index, HF etiology, and INTERMACS level, resulting in 27 matched pairs in the two groups (Fig. 1).

### Baseline Characteristics and Perioperative Outcomes in Propensity Score (PS) Match Analysis

PS preoperative attributes revealed a higher number of ICD implantation and history of stroke for group 2. Interestingly, patients in group 2 had significantly lower pulmonary artery pressure (PAP) and precapillary wedge pressure (PCWP) in comparison to group 1 (Table 1).

All intraoperative characteristics are summarized in Table 2. All LVAD implantations, but one group 2 patient, were performed via median sternotomy. Concomitant surgical procedures were uniformly performed in both groups, however with an expectedly higher rate of atrial septal defect closures in group 2 because of TMR (*p* < 0.001). Temporary mechanical circulatory support (MCS) for perioperative right heart failure was required in *n* = 8 patients (30%) in group 1 and in *n* = 7 patients (26%) in group 2 (*p* > 0.05). The 30-day mortality rate was *n* = 1 patient (3.7%) in group 1 and *n* = 2 patients (7.4%) in group 2, respectively, and all these patients needed temporary MCS for perioperative right heart failure. Causes of death were septic shock and multi-organ failure.

**Table 2:** Procedural characteristics of PS-matched cohort; *n* (%) if not otherwise specified

**Perioperative outcomes in PS-matched cohort (*n* = 27)**			
	HM3 + FMR (*n* = 27)	HM3 + TMR (*n* = 27)	*p* value
Time frame between TMR implantation and LVAD implantation	-	26 months (3.5–26; range, 0–85)	-
**Surgical access**			
Median sternotomy	27 (100)	26 (96)	1
Lateral thoracotomy	0 (0)	1 (4)	1
**Additional surgical procedure during LVAD implantation**			
Atrial septum defect closure	2 (7.4)	16 (59)	< 0.001
Aortic valve replacement	2 (7.4)	4 (15)	0.67
Tricuspid valve repair	3 (11)	9 (33)	0.099
Ascending aorta replacement	0 (0)	1 (4)	1
Left atrial appendage closure	0 (0)	4 (15)	0.11
**Perioperative outcomes**			
CentriMag implantation	8 (30)	7 (26)	1
Mortality within 30 days	1 (4, sepsis)	2 (7.4, MOF)	1

*HM*, HeartMate 3; *TMR*, transcatheter percutaneous mitral valve repair; *LVAD*, left ventricular assist device; *MOF*, multiple organ failure.

### Invasive Hemodynamics After LVAD Implantation

**Fig. 2:**
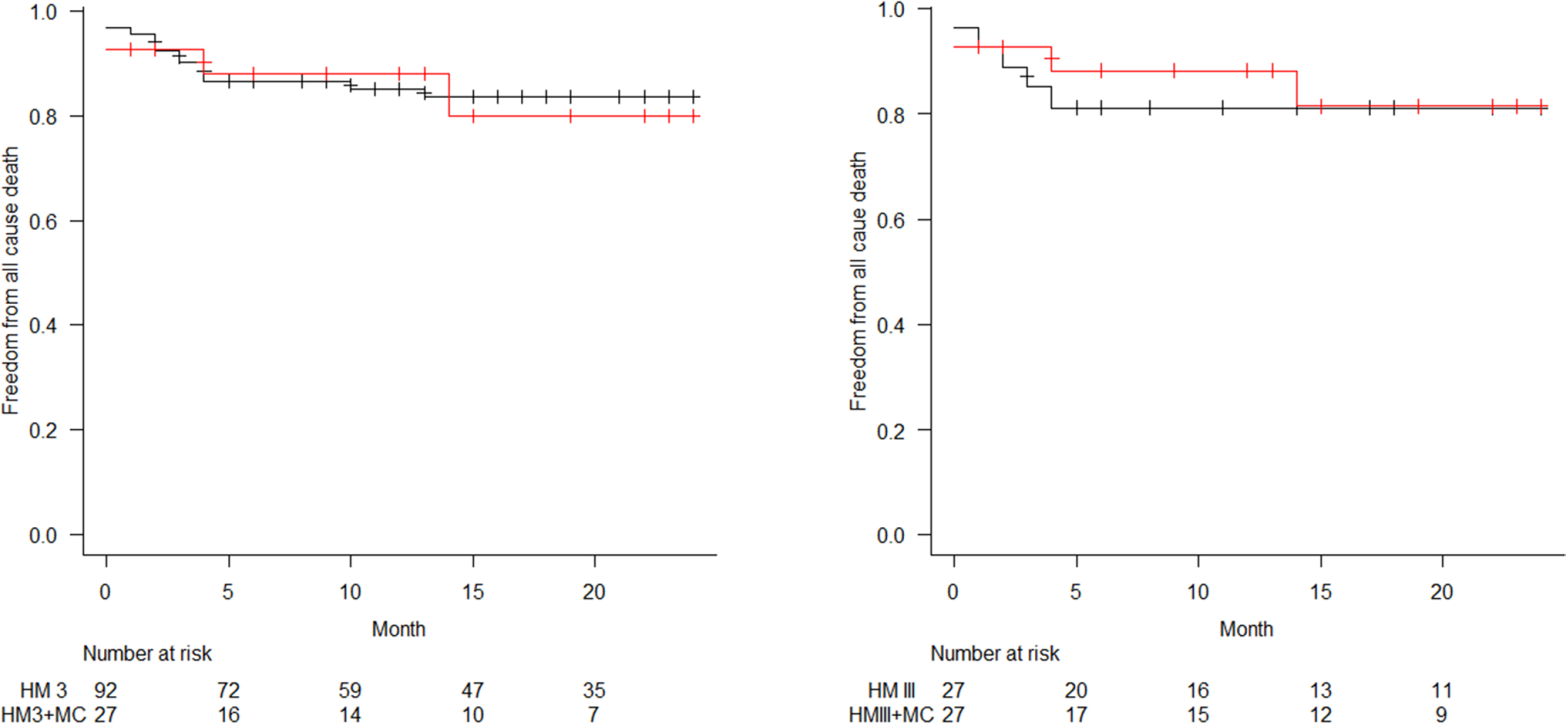
Kaplan–Meier survival analysis

**Fig. 3:**
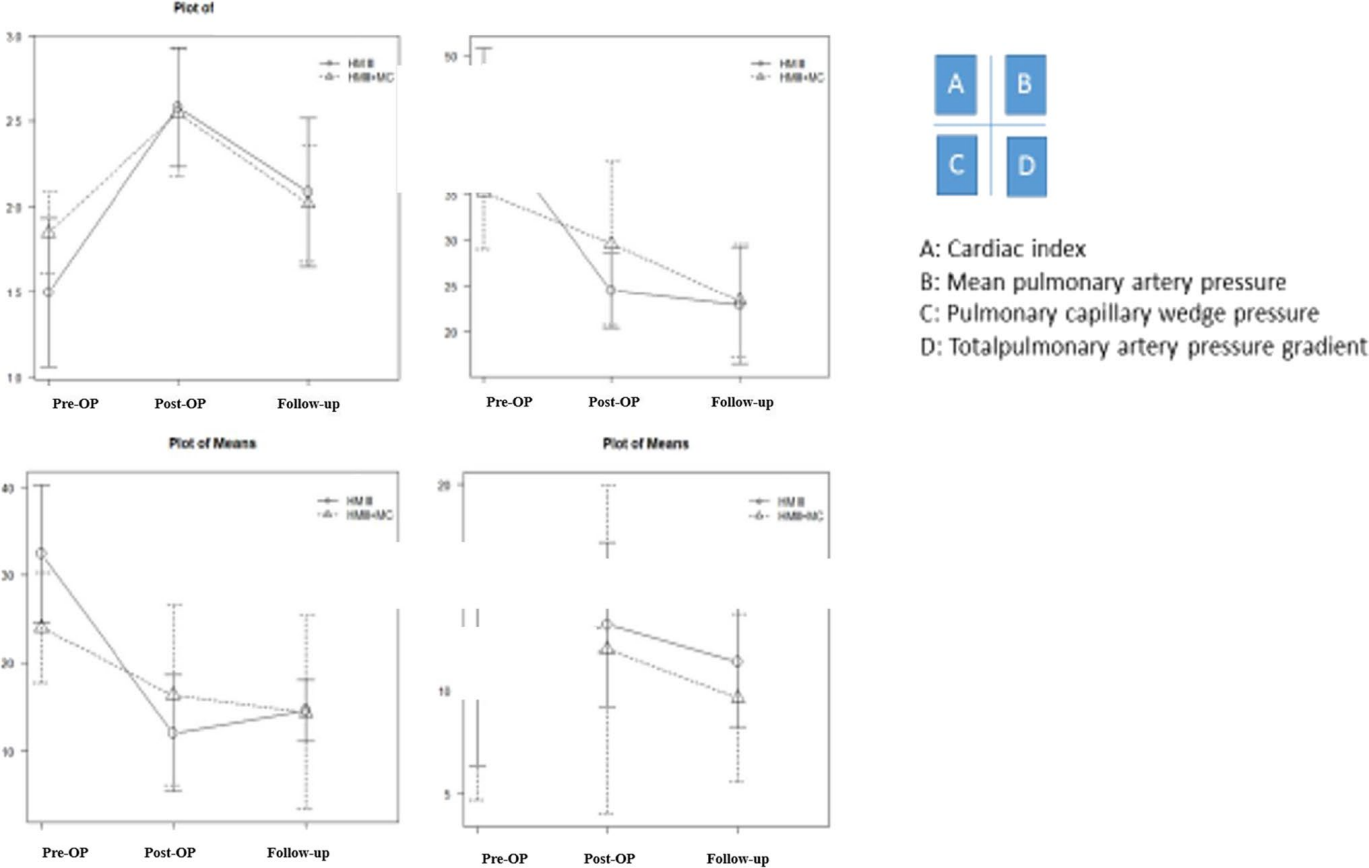
Comparison of invasive hemodynamic measures before, after LVAD implantation (right heart catheter), and at follow-up. There was no statistical difference at each time (preoperative, postoperative, and follow-up) between HM3 and HM3 + MC group. HM3, HeartMate 3; MC, MitraClip

In PS-matched analysis, pulmonary artery pressure (PAP) and pulmonary capillary wedge pressure (PCWP) significantly declined in both groups in comparison to preoperative measures (Tables 1 and 2). LVAD implantation was associated with a significant increase of cardiac index (CI) (Tables 1 and 2), and invasive hemodynamic parameters were not statistically significantly different between the two groups at the time of discharge (Fig. 2). After a mean follow-up of 15 months, invasive PAP and PCWP were in normal range in most patients (Table 4), while transpulmonary pressure gradients were lower in TMR patients, which was not statistically significantly different between the two groups (Fig. 3). This aspect is of particular importance in the context of heart transplantation.

### Mid-term Survival and Predictors of All-Cause Mortality After 2 Years of Follow-up

Two-year survival after LVAD implantation was 83.5% (95%CI 73.6–90%) in group 1 and 80% (95%CI 52.4–92.6%) in group 2, respectively (*p* = 0.87). No baseline characteristic reached statistical significance regarding survival prediction between the two groups during the 2-year follow-up (Fig. 2). Similarly, in the PS-matched cohort, 2-year survival after LVAD implantation was 81.1% (95%CI 60.4–91.7%) in group 1 and 80% (95%CI 52.4–92.6%) in group 2 (*p* = 0.84), while patients that had received heart transplantation were censored in the follow-up analysis (Table 3).

**Table 3:** Cox regression model analysis at 2-year follow-up

	Hazard ratio TMR group	95%CI	*p* value	Hazard ratio FMR group	95%CI	*p* value
Complete cohort						
Total bilirubin	1.07	0.88–1.30	0.48			
CentriMag use	4.55	1.78–11.64	0.0015	4.61	1.81–11.70	0.0013
MitraClip use	1.36	0.44–4.20	0.59			
Matched cohort						
Total bilirubin	1.53	0.95–2.46	0.07763	1.79	1.19–2.69	0.0051
CentriMag use	2.49	0.52–12.02	0.25			
MitraClip use	1.06	0.28–4.10	0.93			

Severe adverse events in group 1 consisted of one patient suffering from therapy refractory right heart failure, another patient had gastrointestinal (GI) bleeding, and one patient developed LVAD pump infection. All three group 1 patients received urgent heart transplantation at 2, 3, and 22 months after LVAD implantation. In group 2, one patient developed progressive right heart failure and one suffered from substantial GI bleeding, and these two group 2 patients received urgent heart transplantation at 1.5 and 13 months after LVAD implantation.

During follow-up, no MitraClip-associated complication was observed, except for relative mitral valve stenosis with a peak transvalvular pressure gradient of 7.5 mmHg in one patient without clinical correspondence. Multivariable Cox regression analysis after 2 years in the unmatched cohort identified postoperative temporary MCS for perioperative right heart failure to represent an independent risk factor for mortality (HR 4.6; 95%CI 1.8–11.7, *p* = 0.0013). In PS-matched analysis, only preoperative total bilirubin level remained as an independent risk factor for mortality (HR 1.8; 95%CI 1.2–2.7, *p* = 0.0051) (Table 4). MitraClip application was not associated with an increased risk in any aspect in this LVAD study population.

**Table 4:** Comparison of preoperative, postoperative, and follow-up values of right heart catheterization of the full cohort and PS-matched cohort. *HM3*, HeartMate 3; *FMR*, functional mitral regurgitation; *TMR*, transcatheter percutaneous mitral valve repair; *CI*, cardiac index; *PAP*, pulmonary artery pressure; *PCWP*, pulmonary capillary wedge pressure; *TPG*, transpulmonary pressure gradient. *p value; preoperative vs. follow-up

	HM3			HM3 + FMR (*n* = 9)			HM3 + TMR		HM3 + TMR (*n* = 6)	
	Preoperative (*n* = 24)	Postoperative (*n* = 22)	*p* value	Follow-up	*p value	Preoperative (*n* = 21)	Postoperative (*n* = 20)	*p* value	Follow-up	*p value
CI, mean ± SD (L/min/m^2^)	1.6 ± 0.48	2.6 ± 0.34	< 0.001	2.1 ± 0.41	0.0084	1.7 + 0.48	2.6 ± 0.31	< 0.001	2.0 ± 0.32	0.44
Mean PAP, mean ± SD (mmHg)	41 ± 9.1	27 ± 9.2	0.0016	23 ± 6.0	< 0.001	34 ± 8.0	30 ± 8.2	0.037	21 + 8.1	0.048
PCWP, mean ± SD (mmHg)	31 ± 7.9	14 ± 7.4	0.0035	13 ± 7.4	0.0015	25 ± 8.2	16 ± 5.8	< 0.001	11 ± 8.4	0.018
TPG, mean ± SD (mmHg)	11 ± 6.3	16 ± 7.1	0.44	10 ± 4.3	0.711	9.4 ± 5.3	15 ± 7.3	0.059	10 ± 3.9	0.93

## Discussion

This is the first study to investigate hemodynamics and safety for the combined use of the two increasingly utilized HF treatments, transcatheter mitral valve repair (TMR), and left ventricular assist device (LVAD) implantation. No study has analyzed the interplay or reciprocal interference of the two therapies yet, and our study further includes invasive pre-, peri-, and post-surgical hemodynamics as well as a 2-year follow-up mortality analysis. In addition, this study evaluated TMR impact on perioperative complications during LVAD implantation and investigated all interactions during the 2-year follow-up.

We found no statistically significant interplay of TMR in patients undergoing LVAD implantation. TMR had no adverse or downside effect in our study, not even through propensity score match analysis. Nevertheless, TMR appeared to have beneficial purposes with reduced pulmonary artery pressures and lower pulmonary capillary wedge pressures in the TMR study group.

Mortality after LVAD implantation is comparable in patients with and without TMR. TMR and untreated functional severe mitral valve regurgitation were both predictors of mortality. Only periprocedural right ventricular mechanical support and preoperative bilirubin remained as independent predictors of mortality in this trial.

In line with current literature on durable MCS, our current data indicates that right heart failure and the necessity for temporary MCS trigger adverse outcome [7–9]. Many efforts have been made to identify predictors for peri- and postoperative right heart failure [7–9], but it remains an open question because no parameter has been identified to improve outcome in LVAD right heart failure so far and cardiac index decline during follow-up in our study population may particularly represent right heart failure [10, 11]. No predictor for right heart failure is available in LVAD patients, neither for worsening of preexisting right heart failure, not for postprocedural right heart failure development [12].

Inclusion of additional HF comorbidities is essential in the context of mortality, and liver dysfunction has been acknowledged as a clear risk factor for outcome before [13], and our study results are unconflicted with the current literature by showing preoperative bilirubin as a robust independent predictor of mortality after LVAD implantation.

For the lively discussion whether patients with end-stage HF would benefit from TMR in conjunction with the necessity of LVAD implantation, our study now demonstrates not only endured TMR efficacy and safety for the combination of TMR and LVAD with no relevant complications in this setting during a 2-year follow-up period, but we can also show potential benefit for combining TMR and LVAD, because our study group of TMR and LVAD had reduced pulmonary artery pressures and lower pulmonary capillary wedge pressures, which is of importance when it comes to heart transplantation [14]. Combining TMR and LVAD may therefore allow heart transplantation in patients that otherwise have a contraindication for increased pulmonary artery and pulmonary capillary wedge pressures. One could therefore argue that TMR may be an option to bridge end-stage HF patients with FMR to heart transplantation, because the development of excessive secondary pulmonary hypertension and pulmonary vascular resistance are contraindications for heart transplantation [15]. Moreover, perioperative pulmonary hypertension has also been suggested to trigger postoperative right heart failure in LVAD [14].

Hereafter, our study does not confirm previous findings indicating that TMR would negatively interfere with LVAD performance [6, 16]. In our study trans-mitral valve pressure gradients after TMR did not exceed 4 mmHg under LVAD, even when 3 clips were implanted, and only one patient revealed a trans-mitral valve gradient of 7.5 mmHg, which was clinically inert on LVAD.

Nevertheless, our study design does not allow to draw conclusions whether TMR could accelerate HF progression in LVAD as suggested before [6], questions that clearly require future prospective investigation. This aspect is under intense discussion currently, because it remains to be elucidated whether the reduction of right ventricular afterload based on scaled mitral valve regurgitation through clip application is of help or whether simply the intra-atrial shunt through iatrogenic atrial septal defect after TMR facilitates beneficial effect [17]. In fact, controlled atrial septostomy may be considered as a palliation in the treatment of pulmonary hypertension [17].

Our study results add to the present controversial discussion on whether severe functional mitral regurgitation requires treatment in patients eligible for LVAD implantation [18]. We cannot demonstrate benefit of TMR in the context of outcome after LVAD implantation, but most of our study patients are classified emergency corresponding as INTERMACS level 1 or 2.

Interestingly, survival after LVAD implantation is better in our trial (81.4%) than reported in the COAPT trial after TMR (71.8%) [4], but the combination has hardly been studied.

A small case series of Ammirati et al. describes six patients with TMR undergoing LVAD implantation, and two patients in that study underwent subsequent heart transplantation [19]. Three patients died 3 months after implantation for right heart failure, one patient died after 13 months because of major stroke, and one patient died after 3 years because of sepsis [19]. With only one patient alive in this report, it is impossible to draw any conclusion [19]. Our study reveals a 2-year survival above 80% both in the TMR + HM3 and the FMR + HM3 group. Although tear, degeneration, and increased infection rates of the mitral valve are known to be TMR associated complications [20], there was no such complication observed during follow-up in our study population.

When not only regarding mortality in this exquisitely vulnerable end-stage HF patient population, we identified right heart failure with indication of temporary right ventricular mechanical support, as well as perioperative bilirubin to predict outcome, but not TMR, clearly representing the complexity of accompanied comorbidities in these patients [21–23].

Therefore, more investigations are needed to elucidate optimized treatment strategies for both end-stage heart failure and functional mitral valve regurgitation, with taking notice of important accompanying comorbidities in this growing and scarcely studied patient population.

### Study Limitations

This is a single-center observational study with a limited number of patients. Due to the design of our study, it may miss confounding factors that could potentially have influenced our results. Thus, conclusions from our study should be interpreted with caution until confirmed by large prospective and randomized clinical trials.

## Conclusion

In patients with TMR, HeartMate 3 LVAD implantation was safe and not affecting LVAD function as well as adverse events such as mortality in a 2-year follow-up, while TMR significantly improved right heart hemodynamics. Temporary right ventricular mechanical support is a predictor of mortality, while in propensity score match analysis, preoperative bilirubin is the only remaining predictor of mortality in this patient population.

## Abbreviations

**FMR**: Functional severe mitral regurgitation
**HF**: Heart failure
TMR: Transcatheter percutaneous edge-to-edge mitral valve repair
LVAD: Left ventricular assist device
TEE: Transesophageal echocardiography
MCS: Mechanical circulatory support

## References

[1] Goliasch G, Bartko PE, Pavo N, Neuhold S, Wurm R, Mascherbauer J, Lang IM, Strunk G, Hulsmann M (2018). Refining the prognostic impact of functional mitral regurgitation in chronic heart failure. European Heart Journal.

[2] Coats AJS, Anker SD, Baumbach A, Alfieri O, von Bardeleben RS, Bauersachs J, Bax JJ, Boveda S, Celutkiene J, Cleland JG, Dagres N, Deneke T, Farmakis D, Filippatos G, Hausleiter J, Hindricks G, Jankowska EA, Lainscak M, Leclercq C, Lund LH, McDonagh T, Mehra MR, Metra M, Mewton N, Mueller C, Mullens W, Muneretto C, Obadia JF, Ponikowski P, Praz F, Rudolph V, Ruschitzka F, Vahanian A, Windecker S, Zamorano JL, Edvardsen T, Heidbuchel H, Seferovic PM, Prendergast B (2021). The management of secondary mitral regurgitation in patients with heart failure: A joint position statement from the Heart Failure Association (HFA), European Association of Cardiovascular Imaging (EACVI), European Heart Rhythm Association (EHRA), and European Association of Percutaneous Cardiovascular Interventions (EAPCI) of the ESC. European Heart Journal.

[3] Friedrichs K, Rudolph V (2019). MITRA-FR and COAPT: Why are the results so different and what are the consequences for the daily routine?. Herz.

[4] Stone GW, Lindenfeld J, Abraham WT, Kar S, Lim DS, Mishell JM, Whisenant B, Grayburn PA, Rinaldi M, Kapadia SR, Rajagopal V, Sarembock IJ, Brieke A, Marx SO, Cohen DJ, Weissman NJ, Mack MJ, Investigators C (2018). Transcatheter mitral-valve repair in patients with heart failure. New England Journal of Medicine.

[5] Kreusser MM, Hamed S, Weber A, Schmack B, Volz MJ, Geis NA, Grossekettler L, Pleger ST, Ruhparwar A, Katus HA, Raake PW (2020). MitraClip implantation followed by insertion of a left ventricular assist device in patients with advanced heart failure. ESC Heart Fail.

[6] Dogan G, Hanke JS, Ricklefs M, Chatterjee A, Feldmann C, Mashaqi B, Deniz E, Haverich A, Schmitto JD (2018). MitraClip procedure prior to left ventricular assist device implantation. J Thorac Dis.

[7] Gummert JF, Haverich A, Schmitto JD, Potapov E, Schramm R, Falk V (2019). Permanent implantable cardiac support systems. Deutsches Ärzteblatt International.

[8] Schramm R, Zittermann A, Morshuis M, Schoenbrodt M, von Roessing E, von Dossow V, Koster A, Fox H, Hakim-Meibodi K, Gummert JF (2020). Comparing short-term outcome after implantation of the HeartWare(R) HVAD(R) and the Abbott(R) HeartMate 3(R). ESC Heart Fail.

[9] Hrytsyna Y, Kneissler S, Kaufmann F, Muller M, Schoenrath F, Mulzer J, Sundermann SH, Falk V, Potapov E, Knierim J (2021). Experience with a standardized protocol to predict successful explantation of left ventricular assist devices. Journal of Thoracic and Cardiovascular Surgery.

[10] Konstam MA, Kiernan MS, Bernstein D, Bozkurt B, Jacob M, Kapur NK, Kociol RD, Lewis EF, Mehra MR, Pagani FD, Raval AN, Ward C, American Heart Association Council on Clinical, C., Council on Cardiovascular Disease in the, Y., Council on Cardiovascular, S., & Anesthesia (2018). Evaluation and management of right-sided heart failure: A scientific statement from the American Heart Association. Circulation.

[11] Kassner A, Oezpeker C, Gummert J, Zittermann A, Gartner A, Tiesmeier J, Fox H, Morshuis M, Milting H (2021). Mechanical circulatory support does not reduce advanced myocardial fibrosis in patients with end-stage heart failure. European Journal of Heart Failure.

[12] Soliman OII, Akin S, Muslem R, Boersma E, Manintveld OC, Krabatsch T, Gummert JF, de By T, Bogers A, Zijlstra F, Mohacsi P, Caliskan K, Investigators E (2018). Derivation and validation of a novel right-sided heart failure model after implantation of continuous flow left ventricular assist devices: The EUROMACS (European Registry for Patients with Mechanical Circulatory Support) right-sided heart failure risk score. Circulation.

[13] Farag M, Veres G, Szabo G, Ruhparwar A, Karck M, Arif R (2019). Hyperbilirubinaemia after cardiac surgery: The point of no return. ESC Heart Fail.

[14] Biner S, Siegel RJ, Feldman T, Rafique AM, Trento A, Whitlow P, Rogers J, Moon M, Lindman B, Zajarias A, Glower D, Kar S, investigators, E (2012). Acute effect of percutaneous MitraClip therapy in patients with haemodynamic decompensation. European Journal of Heart Failure.

[15] Mehra MR, Canter CE, Hannan MM, Semigran MJ, Uber PA, Baran DA, Danziger-Isakov L, Kirklin JK, Kirk R, Kushwaha SS, Lund LH, Potena L, Ross HJ, Taylor DO, Verschuuren EAM, Zuckermann A, International Society for Heart Lung Transplantation Infectious Diseases, P., Heart, F., & Transplantation, C (2016). The 2016 International Society for Heart Lung Transplantation listing criteria for heart transplantation: A 10-year update. J Heart Lung Transplant.

[16] Mehra MR, Uriel N, Naka Y, Cleveland JC, Yuzefpolskaya M, Salerno CT, Walsh MN, Milano CA, Patel CB, Hutchins SW, Ransom J, Ewald GA, Itoh A, Raval NY, Silvestry SC, Cogswell R, John R, Bhimaraj A, Bruckner BA, Lowes BD, Um JY, Jeevanandam V, Sayer G, Mangi AA, Molina EJ, Sheikh F, Aaronson K, Pagani FD, Cotts WG, Tatooles AJ, Babu A, Chomsky D, Katz JN, Tessmann PB, Dean D, Krishnamoorthy A, Chuang J, Topuria I, Sood P, Goldstein DJ, Investigators M (2019). A fully magnetically levitated left ventricular assist device - Final report. New England Journal of Medicine.

[17] Berry N, Mauri L, Feldman T, Komtebedde J, van Veldhuisen DJ, Solomon SD, Massaro JM, Shah SJ (2020). Transcatheter InterAtrial Shunt Device for the treatment of heart failure: Rationale and design of the pivotal randomized trial to REDUCE Elevated Left Atrial Pressure in Patients with Heart Failure II (REDUCE LAP-HF II). Am Heart J.

[18] Godino C, Munafo A, Scotti A, Estevez-Loureiro R, Portoles Hernandez A, Arzamendi D, Fernandez Peregrina E, Taramasso M, Fam NP, Ho EC, Asgar A, Vitrella G, Raineri C, Adamo M, Fiorina C, Montalto C, Fraccaro C, Giannini C, Fiorelli F, Popolo Rubbio A, Ooms JF, Compagnone M, Maffeo D, Bettari L, Furholz M, Tamburino C, Petronio AS, Grasso C, Agricola E, Van Mieghem NM, Tarantini G, Curello S, Praz F, Pascual I, Potena L, Colombo A, Maisano F, Metra M, Margonato A, Crimi G, Saia F (2020). MitraClip in secondary mitral regurgitation as a bridge to heart transplantation: 1-year outcomes from the International MitraBridge Registry. Journal of Heart and Lung Transplantation.

[19] Ammirati E, Van De Heyning CM, Musca F, Brambatti M, Perna E, Cipriani M, Cannata A, Mondino M, Moreo A, De Bock D, Pretorius V, Claeys MJ, Adler ED, Russo CF, Frigerio M (2019). Safety of centrifugal left ventricular assist device in patients previously treated with MitraClip system. Int J Cardiol.

[20] Takayuki G, Soren S, Kristin R, Harnath A, Grimmig O, Soren J, Dirk F (2019). Surgical revision of failed percutaneous edge-to-edge mitral valve repair: Lessons learned. Interactive Cardiovascular and Thoracic Surgery.

[21] Gyoten T, Rojas SV, Irimie A, Schramm R, Morshuis M, Gummert JF, Sitzer M, Fox H (2021). Patients with ventricular assist device and cerebral entrapment-Supporting skullcap reimplantation. Artificial Organs.

[22] Ibrahim M, Arafat S, Rojas SV, Schramm R, Gummert JF, Morshuis M, Fox H (2020). Facilitating heart transplantability in an end stage heart failure patient with brain abscess and infected left ventricle assist device A unique case report. International Journal of Surgery Case Reports.

[23] Gyoten T, Morshuis M, Rojas SV, Deutsch MA, Schramm R, Gummert JF, Fox H (2021). Identification of characteristics, risk factors, and predictors of recurrent LVAD thrombosis: Conditions in HeartWare devices. Journal of Artificial Organs.

